# Methylation-mediated silencing and tumour suppressive function of *hsa-miR-124 *in cervical cancer

**DOI:** 10.1186/1476-4598-9-167

**Published:** 2010-06-26

**Authors:** Saskia M Wilting, Robert AA van Boerdonk, Florianne E Henken, Chris JLM Meijer, Begoňa Diosdado, Gerrit A Meijer, Carlos le Sage, Reuven Agami, Peter JF Snijders, Renske DM Steenbergen

**Affiliations:** 1 Department of Pathology, VU University Medical Center, Amsterdam, The Netherlands; 2 Division of Gene Regulation, the Netherlands Cancer Institute, Amsterdam, The Netherlands

## Abstract

**Background:**

A substantial number of microRNAs (miRNAs) is subject to epigenetic silencing in cancer. Although epigenetic silencing of tumour suppressor genes is an important feature of cervical cancer, little is known about epigenetic silencing of miRNAs. Since DNA methylation-based silencing of *hsa-miR-124 *occurs in various human cancers, we studied the frequency and functional effects of *hsa-miR-124 *methylation in cervical carcinogenesis.

**Results:**

Quantitative MSP analysis of all 3 loci encoding the mature *hsa-miR-124 *(hsa-miR-124-1/-2/-3) showed methylation in cervical cancer cell lines SiHa, CaSki and HeLa as well as in late passages of human papillomavirus (HPV) type 16 or 18 immortalised keratinocytes. Treatment of SiHa cells with a demethylating agent reduced *hsa-miR-124 *methylation levels and induced *hsa-miR-124 *expression. In HPV-immortalised keratinocytes increased methylation levels were related to reduced *hsa-miR-124 *expression and higher mRNA expression of *IGFBP7*, a potential *hsa-miR-124 *target gene. Ectopic *hsa-miR-124 *expression in SiHa and CaSki cells decreased proliferation rates and migratory capacity. Combined hsa-miR-124-1 and/or hsa-miR-124-2 methylation analysis of 139 cervical tissue specimens showed an increasing methylation frequency from 0% in normal tissues up to 93% in cervical carcinomas. Increased methylation levels of hsa-miR-124-1 and hsa-miR-124-2 were significantly correlated with reduced *hsa-miR-124 *expression in cervical tissue specimens. Combined hsa-miR-124-1 and/or hsa-miR-124-2 methylation analysis of 43 cervical scrapes of high-risk HPV positive women was predictive of underlying high-grade lesions.

**Conclusions:**

DNA methylation-based silencing of *hsa-miR-124 *is functionally involved in cervical carcinogenesis and may provide a valuable marker for improved detection of cervical cancer and its high-grade precursor lesions.

## Background

Cervical cancer is the second most common cancer in women worldwide and is caused by a persistent infection with high-risk types of the human papillomavirus (hrHPV) [1-3]. The development of cervical squamous cell carcinomas (SCCs, representing about 80% of cases) occurs via well-recognisable premalignant precursor lesions (cervical intraepithelial neoplasia (CIN), graded 1-3), whereas less is known about the different precursor stages preceding cervical adenocarcinomas (AdCAs, accounting for 10-20% of cases).

Even taking the promising results of recently introduced prophylactic HPV vaccines into account, cervical screening will remain necessary in the foreseeable future [4-7]. Recent studies have shown that the sensitivity of hrHPV testing is superior to that of cytology as a screening tool [8-10]. However, hrHPV testing also results in the identification of a considerable number of hrHPV-positive women without (pre)cancerous lesions, necessitating the development of proper triage tests for hrHPV-positive women. Assays detecting (epi)genetic changes that besides hrHPV are crucial for malignant progression will likely contribute to the discrimination of hrHPV positive women with cervical (pre)cancer.

DNA methylation-mediated silencing of an increasing number of protein-coding tumour suppressor genes is known to be involved in cervical cancer. Therefore, the present study aimed to investigate whether epigenetic changes relevant in hrHPV-mediated cervical carcinogenesis may affect the expression microRNAs (miRNAs) as well [11-16]. miRNAs, ~23 nucleotide long, non-coding RNAs, regulate expression of protein-coding genes at the posttranscriptional level by sequence specific base pairing in the 3' untranslated region (UTR) of the target mRNAs. Recent proteomic studies have shown that a single miRNA can regulate expression of hundreds of targets [17,18]. The potential importance of miRNAs in cervical carcinogenesis in general, is underlined by a number of studies. miRNA loci are significantly associated with fragile sites, which are known insertion sites of HPV in cervical cancers. In addition, even though no HPV-encoded miRNAs have been identified so far, HPV-encoded genes were shown to influence the miRNA expression of its host cell [19,20]. Altered miRNA expression was found in cervical cancer cell lines and/or cervical carcinomas compared to normal controls and a number of these differentially expressed miRNAs were shown to influence proliferation rates of cervical cancer cell lines SiHa or HeLa [20-24].

Interestingly, similar to protein-coding tumour suppressor genes, expression of a substantial number of miRNAs was shown to be under epigenetic regulation [25-31]. A well-known epigenetically silenced miRNA in human carcinogenesis is *hsa-miR-124*. DNA methylation of *hsa-miR-124 *was first shown by Lujambio *et al *in colon, breast and lung cancer, as well as in leukaemia and lymphoma [29]. Subsequent studies confirmed frequent *hsa-miR-124 *methylation in leukaemia affecting clinical outcome and additionally showed frequent *hsa-miR-124 *methylation in gastric cancer and hepatocellular carcinoma [25,26,30,32]. At present, no studies have been performed to investigate the role of epigenetic silencing of miRNAs in cervical cancer.

In this study we evaluated the potential role of DNA methylation-based silencing of *hsa-miR-124 *during cervical carcinogenesis. The mature *hsa-miR-124 *sequence is processed from 3 separate premature sequences, located at chromosomes 8p23.1 (miR-124-1), 8q12.3 (miR-124-2) and 20q13.33 (miR-124-3), all of which contain CpG islands in their promoter regions. We investigated the methylation status of all 3 genomic loci encoding the mature *hsa-miR-124 *in cervical cancer cell lines, a longitudinal *in vitro *model system of hrHPV-induced carcinogenesis [33], cervical tissue specimens (n = 139), and hrHPV-positive cervical scrapes (n = 43) of women with and without a CIN3 diagnosis in follow-up. In addition, effects of (ectopic) *hsa-miR-124 *expression on cellular proliferation, migration and mRNA expression of *IGFBP7*, a potential target gene, were studied.

## Materials and methods

### Cell lines and cell culture

Establishment and culture of the HPV16 (FK16A/FK16B) and HPV18 (FK18A/FK18B) immortalised cell lines have been described previously [33]. Primary human keratinocytes, referred to as EK cells, were isolated from foreskin and cultured as described previously [33]. The human cervical carcinoma cell lines SiHa, CaSki and HeLa were obtained from the American Type Culture Collection (Manassas, VA, USA). SiHa cells were treated with 5000 nM 5-aza-2'-deoxycytidine (DAC, Sigma Chemical Co., St. Louis, MO, USA) dissolved in PBS to analyse the effect of global methylation inhibition on *hsa-miR-124 *expression.

### Clinical tissue specimens

We used frozen specimens of normal cervix (n = 5), CIN2/3 (n = 7), SCC (n = 9) and AdCA (n = 5) as well as formalin-fixed, paraffin-embedded (FFPE) biopsy specimens of normal cervix (n = 18), CIN1 (n = 36), CIN3 (n = 41), SCC (n = 29) and AdCA (n = 15). All specimens were collected during the course of routine clinical practice and stored at the Department of Pathology at the VU University Medical Center (Amsterdam, the Netherlands). Normal specimens were obtained from non-cancer patients undergoing hysterectomy. The mean age of all women included in this study was 40.3 years (range 18-79). Per histological subgroup the women had the following mean ages: 49.1 years (range 34-70) in the normal group; 35.9 (range 22-52) years in the CIN1 group; 35.9 years (range 27-74) in the CIN3 group; 53.0 years (range 35-61) in the SCC group; 45.6 years (range 28-79) in the AdCA group. The mean age in any of the groups of women with cervical (pre)malignant disease was not significantly higher than that of the women with normal histology.

Cervical scrapings were obtained from the population-based cervical screening trial POBASCAM, registered as an International Standard Randomized Controlled Trial under number ISRCTN20781131 [8,34]. For this study, we selected 22 cervical scrapes from hrHPV-positive women who had normal cytology without evidence of CIN disease up to the next screening round (i.e. 5 years) and 21 scrapings classified as severe dyskaryosis or worse from hrHPV-positive women who had a CIN3 diagnosis within 18 months of follow-up. The mean age of the women with normal cytology without CIN disease was 33.2 years (range 18-53), and that of women with abnormal cytology with CIN3 was 35.14 years (range 25-55). This study followed the ethical guidelines of the Institutional Review Board of the VU University Medical Center.

### Extraction of nucleic acids and HPV testing

For methylation analysis, DNA was extracted from FFPE specimens by proteinase K digestion and purified using the High Pure PCR Template Preparation Kit (Roche Diagnostics, Almere, The Netherlands) following the manufacturer's recommendations. Genomic DNA from cell lines and frozen specimens was extracted by proteinase K digestion followed by standard phenol-chloroform extraction as described previously [35].

For *hsa-miR-124 *expression analysis, frozen specimens of normal cervix, CIN2/3 lesions, SCCs, and AdCAs were first enriched for epithelial cells by means of laser capture microdissection using a Leica ASLMD microscope (Leica, Heidelberg, Germany) as described before [36]. Subsequently, total RNA was isolated from these samples and cell lines using TRIzol reagent (Life Technologies, Breda, The Netherlands), according to the manufacturer's instructions.

HPV typing of clinical specimens was performed using the general primer GP5+/6+ PCR, followed by reverse line blot, as described previously [37,38] (Additional file 1).

### DNA modification and quantitative methylation-specific PCR (qMSP) analysis

The DNA methylation status of the CpG-island containing promoter regions associated with the three genomic loci encoding *hsa-miR-124 *(hsa-miR-124-1, hsa-miR-124-2 and hsa-miR-124-3) was determined by qMSP analysis on sodium bisulfite-treated genomic DNA from cell lines, tissue specimens and scrapings. In brief, genomic DNA was modified using the EZ DNA Methylation kit (Zymo Research, Orange, CA, USA), which induces chemical conversion of unmethylated cytosines into uracils, whereas methylated cytosines are protected from this conversion. Specific primers were designed to amplify the methylated DNA sequence of all 3 promoter regions. Amplicons (hsa-miR-124-1: -191 to -97; hsa-miR-124-2: -301 to -163; hsa-miR-124-3: -106 to -11 relative to the transcription start site, respectively) were detected and quantified using TaqMan probes (Table 1). In addition, the modified, unmethylated sequence of the housekeeping gene β-actin (ACTB) was amplified as a reference [39]. qMSP reactions were carried out in a 12 μl reaction volume containing 50 ng of bisulfite-treated DNA, 417 nM of each primer, 208 nM probe and 1× QuantiTect Probe PCR Kit master mix (Qiagen, Westburg, Leusden, The Netherlands) using the ABI 7500 Fast Real-Time PCR System (Applied Biosystems, Nieuwerkerk a/d IJssel, The Netherlands).

**Table 1:** Sequences of qMSP and qRT-PCR primers used in this study

Gene	Primers(5'-3')	Size (bp)	Annealing (°C)
hsa-miR-124-1	F: CGGCGGGGAGGATGTT		58.9
	R: ATAAAAAACGACGCGTATACGTACG	94	59.4
	P: CGGCGTTTTTTATTTTT-Xsprobe		70.0
hsa-miR-124-2	F: GGGTAATTAATTTGGATTTACGTCGTTAT		59.9
	R: CGTAAAAATATAAACGATACGTATACCTACGT	138	58.8
	P: TTTACAACACACGCCTAAA -Xsprobe		69.0
hsa-miR-124-3	F: ACGCGGCGAAGACGTTT		59.0
	R: CGAACGACGAACGTCGAAA	95	59.4
	P: AAAATCCTCGCCCGAAAAACGCGA		70.4

IGFBP7	F: CCCAGGTGTACTTGAGCTGTGA	89	58.9
	R: TGAACTCCATAGTGACCCCTTTTT		58.9
snRNP U1A	F: TCCTCACCAACCTGCCAGA	71	58.8
	R: TGAAGCCAGGGAACTGATTGA		59.3

F: forward; R: reverse; P: probe; Xsprobe: minor groove binder probe

All PCR experiments were performed in duplicate (delta Ct ≤1.5 between replicates) and mean values were used for calculations. Methylation values of the 3 target regions were normalised to the reference gene ACTB using the comparative Ct method (2^-ΔCT^) [40]. All methylation negative samples in our study had a Ct for ACTB below 32, indicating sufficient DNA quality and thereby excluding false negative results. To ensure the detection of distinguishable increases in methylation level in (pre)malignant cervical lesions over normal cervical controls, we used the 99% confidence interval of the methylation levels obtained in normal cervical controls as cut-off value. Samples above this threshold were considered positive for methylation. For tissue specimens and scrapings separate cut-off values were determined using the appropriate normal controls.

### Retroviral transduction

Retroviral *hsa-miR-124 *or empty vector (ctrl) constructs previously described by Voorhoeve *et al *[41] were transfected into the Phoenix A retrovirus producer cell line and supernatants containing the replication-deficient *hsa-miR-124*-expressing retrovirus or empty vector retrovirus were harvested 48 hours post-transfection. For the transduction experiments, SiHa and CaSki cells were incubated for 16 hours at 37°C with filtered viral supernatants supplemented with polybrene (15 μg/ml). SiHa/CaSki_miR-124 cells or SiHa/CaSki_ctrl cells were selected by continuous culturing of the transduced cells in the presence of blasticidin (3 μg/ml).

### Quantitative Reverse Transcription-PCR (qRT-PCR)

Expression of *hsa-miR-124 *was measured using TaqMan microRNA assays following the manufacturer's instructions (00046 and 001182; Applied Biosystems) on the ABI 7500 Fast Real-Time PCR System (Applied Biosystems). The small nucleolar RNA transcript RNU43 was included as internal reference for *hsa-miR-124 *expression (001095; Applied Biosystems). *Hsa-miR-124 *expression values were normalised to the reference again by using the comparative Ct method as described above.

Intron-flanking primers for *IGFBP7*, a potential target gene of *hsa-miR-124*, were selected using Primer Express 3.0 (Applied Biosystems) (Table 1). Total RNA was reverse transcribed using the specific reverse primer and the resulting cDNA was used for real-time PCR. cDNA corresponding to 25 ng of total RNA was amplified in a total reaction volume of 25 μl containing 12.5 μl 2x Sybr Green master mix (Perkin Elmer/Applied Biosystems) and 0.5 μM primers. The house keeping gene U1 small nuclear ribonucleoprotein specific A protein (*snRNP U1A*) was included as internal reference (Table 1). Expression values of *IGFBP7 *were normalized to this reference by using the comparative Ct method as described above.

### Cellular proliferation and migration assays

Cell proliferation was measured using a colorimetric (MTT-tetrazolium) assay (ICN Biomedicals Inc, OH, USA). In this assay the amount of dye conversion, as measured by the optical density at a wavelength of 540 nm, is directly related to the number of viable cells in each well. In brief, 5000 cells (CaSki) or 10000 cells (SiHa) were seeded in triplicate in 96-well plates and assayed for MTT conversion at day 0, day 1, day 2 and day 5 for CaSki and day 0, day 2, day 3 and day 6 for SiHa. The proliferation rate was determined by subtracting the measurement of day 0 from all other time points.

Cellular migration *in vitro *was determined using a so-called wound-healing assay. Cells were grown to confluency in 24-well plates and a single linear scratch was made in duplicate for all conditions using a sterile tip, resulting in a cell-free zone. Photographs of the scratch were taken immediately post-scratching and following 24 hours of incubation at 37°C. After 48 hours cells were fixed with methanol and stained with crystal violet solution.

### Statistical analysis

Proliferation rates between *hsa-miR-124*-expressing cells and control cells were compared using the Student's t test. The frequency of methylation between normal and low-grade (CIN1) lesions on one hand and high-grade (CIN3) lesions and SCCs on the other hand were compared using χ^2^-testing. The difference in *hsa-miR-124 *expression between normal cervical epithelium and CIN2/3 lesions and carcinomas was compared using the Wilcoxon rank test. Linear (Pearson) correlation was determined between *hsa-miR-124 *methylation levels and *hsa-miR-124 *expression.

## Results

### Methylation of *hsa-miR-124 *during hrHPV-mediated transformation *in vitro*

**Figure 1:**
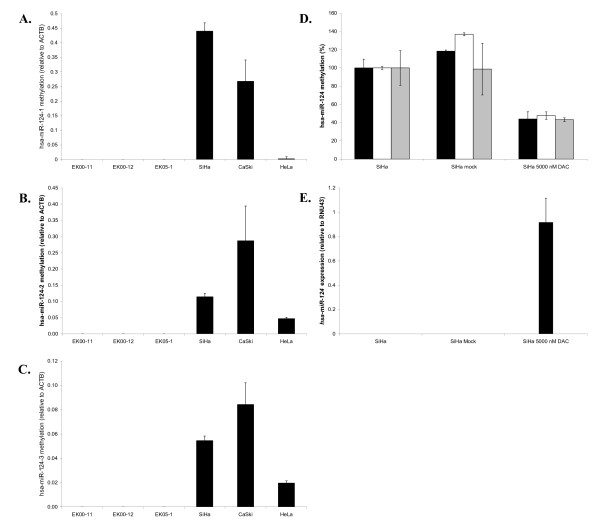
***Hsa-mi*R-124 methylation in primary keratinocytes (EK cells) and cervical cancer cell lines SiHa, CaSki and HeLa. A.** hsa-miR-124-1 methylation, **B.** hsa-miR-124-2 methylation, **C.** hsa-miR-124-3 methylation. Whereas in primary keratinocytes no methylation was detectable, all cervical cancer cell lines were positive for methylation of hsa-miR-124-1, hsa-miR-124-2 and hsa-miR-124-3. **D**. In SiHa cells treated with 5000 nM DAC, methylation levels of hsa-miR-124-1 (black), hsa-miR-124-2 (white) and hsa-miR-124-3 (grey) were reduced by more than 50%. Methylation levels of all regions in untreated cells were set to 100%. **E**. Whereas in untreated and mock (PBS) treated SiHa cells no *hsa-miR-124 *expression was detectable, SiHa cells treated with 5000 nM DAC showed clear *hsa-miR-124 *expression.

To determine whether *hsa-miR-124 *may be silenced due to promoter hypermethylation in cervical cancer, we assessed DNA methylation at the 3 promoter regions of *hsa-miR-124 *(hsa-miR-124-1 located at 8p23.1; hsa-miR-124-2 located at 8q12.3; and hsa-miR-124-3 located at 20q13.33) in cervical cancer cell lines using qMSP analysis. Methylation of all 3 promoter regions of *hsa-miR-124 *was observed in SiHa cells as well as in another cervical cancer cell line, CaSki. A third cervical cancer cell line, HeLa, showed lower levels of methylation for all 3 regions and especially methylation levels of hsa-miR-124-1 were extremely low compared to SiHa and CaSki. Primary keratinocytes isolated from 3 independent donors, on the other hand, were completely unmethylated at all 3 regions (Figure 1A-C). Treatment of the cervical cancer cell line SiHa with the demethylating agent DAC resulted in > 50% decrease of methylation levels at all 3 regions and increased expression of *hsa-miR-124 *compared to untreated and mock-treated SiHa cells (PBS) (Figure 1D and 1E).

**Figure 2:**
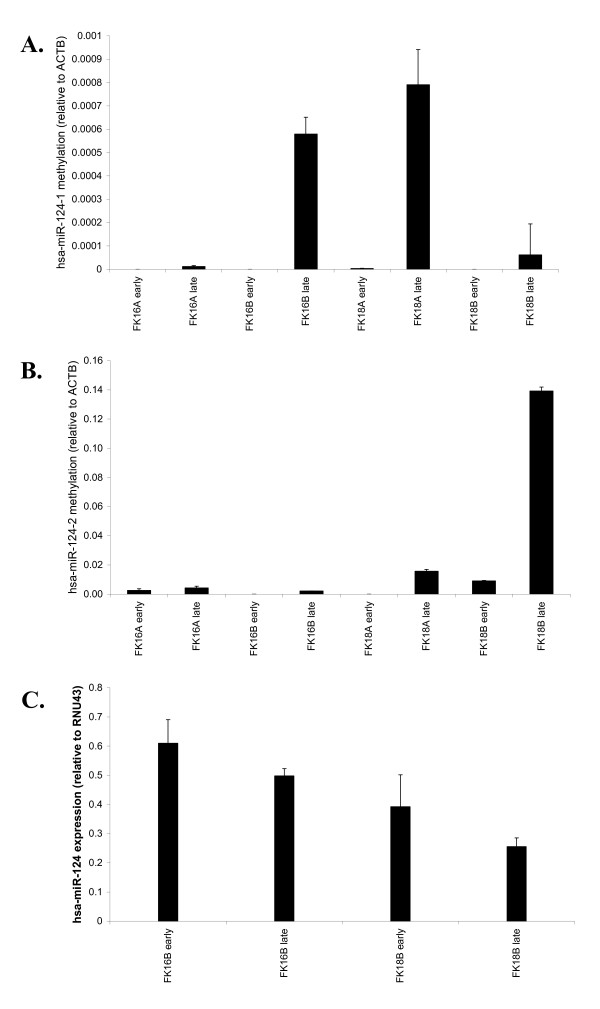
***Hsa-miR-124* methylation and expression in early and late passages of HPV16 (FK16A/FK16B) and 18 (FK18A/FK18B) immortalised keratinocytes. A.** hsa-miR-124-1, **B.** hsa-miR-124-2. Both hsa-miR124-1 and hsa-miR-124-2 showed little to no methylation in early passages and increasing levels of methylation in later passages of HPV16 and HPV18 immortalised cells.  Note the difference in scales of Figure 2A and 2B. Levels of hsa-miR-124-1 methylation in HPV-immortalised keratinocytes are very low compared to the levels seen in cervical cancer cell lines (Figure 1A). No methylation of hsa-miR-124-3 was found in HPV-immortalised keratinocytes. **C**. Late passages of FK16B and FK18B cells showed reduced expression of *hsa-miR-124 *compared to their corresponding earlier passages.

To investigate at which stage during hrHPV-mediated transformation *hsa-miR-124 *becomes methylated we subsequently performed qMSP analysis for all 3 regions on early and late passages of HPV16 (FK16A and FK16B) and HPV18 (FK18A and FK18B) immortalised keratinocyte cell lines. Whereas early passages (range: p23-p43) of all 4 cell lines showed little to no methylation, increased methylation of hsa-miR-124-1 and hsa-miR-124-2 was observed in late passages (range: p70-p96) (Figure 2A and 2B). For hsa-miR-124-1 an increase in methylation levels was observed in late passages of FK16B and FK18A cells. However, the levels of methylation were still very low compared to those observed in SiHa and CaSki cells (Figure 1A). For hsa-miR-124-2, late passages of FK18A and FK18B cells showed increased methylation, of which the methylation level in late passage FK18B cells was comparable to that observed in SiHa cells (Figure 1B). Methylation of the hsa-miR-124-3 region was not detected in any of the HPV-immortalised keratinocytes (data not shown). The increase in hsa-miR-124-1 methylation in late passage FK16B cells, though being quite low compared to SiHa and CaSki cells, was associated with reduced *hsa-miR-124 *expression compared to its corresponding earlier passage (Figure 2C). Similarly, in late passage FK18B cells, showing methylation levels for hsa-miR-124-2 comparable to SiHa cells, lower *hsa-miR-124 *expression was found compared to its earlier passage. Together, these results further support a (direct) correlation between methylation and expression of *hsa-miR-124*. Methylation-mediated silencing of *hsa-miR-124 *appears to occur during HPV-induced carcinogenesis at the post-immortalisation stage and is not directly related to the presence of hrHPV.

### Tumour suppressive activities of *hsa-miR-124* in cervical cancer cell lines

**Figure 3:**
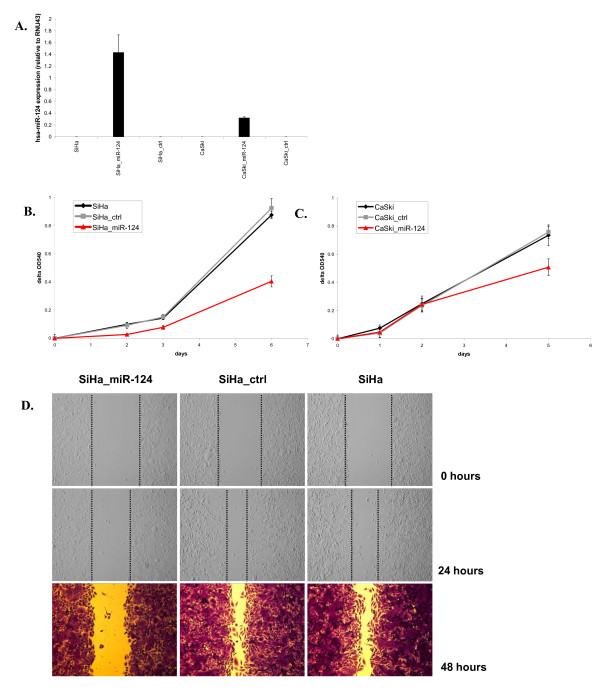
**Ectopic expression of *hsa-miR-124* in SiHa and CaSki cells. A.** Whereas parental cell lines and empty vector control cells (SiHa_ctrl and CaSki_ctrl) showed no detectable expression of *hsa-miR-124*, cells transduced with *hsa-miR-124 *(SiHa_miR-124 and CaSki_miR-124) expressed *hsa-miR-124*. Ectopic *hsa-miR-124 *expression resulted in decreased proliferation rates of **B**. SiHa_miR-124 (red) and **C**. CaSki_miR-124 (red) compared to parental (black) and empty vector control cells (grey). In **D**. results of wound-healing assays in SiHa_miR-124, SiHa_ctrl and SiHa cells are shown, indicating decreased migratory capacity in cells expressing *hsa-miR-124*.

The fact that methylation of *hsa-miR-124 *is consistently found in cervical cancer cell lines and appears to be associated with gene silencing, suggests that *hsa-miR-124 *may possess tumour suppressive traits in cervical cancer. To test this hypothesis, we stably transduced SiHa and CaSki cells with a retroviral *hsa-miR-124*-containing vector (SiHa_miR-124 and CaSki_miR-124, respectively) and an empty vector (SiHa_ctrl and CaSki_ctrl, respectively). Ectopic expression of *hsa-miR-124 *was confirmed by qRT-PCR (Figure 3A). To determine the effects of ectopic *hsa-miR-124 *expression on proliferation we measured cell viability using the MTT assay. The proliferation rate was significantly lower in SiHa_miR-124 compared to SiHa_ctrl cells and parental SiHa cells (Figure 3B; p < 0.01). In CaSki cells effects were less pronounced, but proliferation rates were still significantly lower in CaSki_miR-124 compared to both CaSki_ctrl and CaSki parental cells (Figure 3C; p < 0.05). The somewhat reduced effect observed in CaSki_miR-124 cells compared to SiHa_miR-124 cells is likely correlated to the levels of *hsa-miR-124 *overexpression (Figure 3A).

To measure effects of *hsa-miR-124 *expression on the migratory capacity of SiHa cells we performed a wound-healing assay. Duplicate experiments consistently showed decreased migratory capacity of SiHa_miR-124 compared to SiHa_ctrl cells and parental SiHa cells at 24 and 48 hours (Figure 3D).

In conclusion, these results suggest that ectopic expression of *hsa-miR-124 *in cervical cancer cells has tumour suppressive effects.

### *IGFBP7 *is a potential target of *hsa-miR-124 *in a subset of cervical cancers

To identify potential *hsa-miR-124 *target genes in cervical cancer we used data from a recent study by Baek *et al*, in which the impact of *hsa-miR-124 *expression in HeLa cells on mRNA and protein output was determined [17]. Interestingly, they found that for targets undergoing robust (>1.5 fold) repression, the major mechanism of repression was mRNA destabilisation. Therefore we compared their proteomics data on HeLa cells ectopically expressing *hsa-miR-124 *to our own dataset of genome-wide mRNA expression in cervical SCCs, all of which showed *hsa-miR-124 *methylation by qMSP analysis (data not shown) [36]. This comparison highlighted 1 gene containing an *hsa-miR-124 *target site, namely *IGFBP7*, that showed 3.7 fold protein downregulation in HeLa_miR124 cells and a significant (false discovery rate (FDR) = 0.036) mRNA upregulation in cervical SCCs displaying *hsa-miR-124 *methylation.

**Figure 4:**
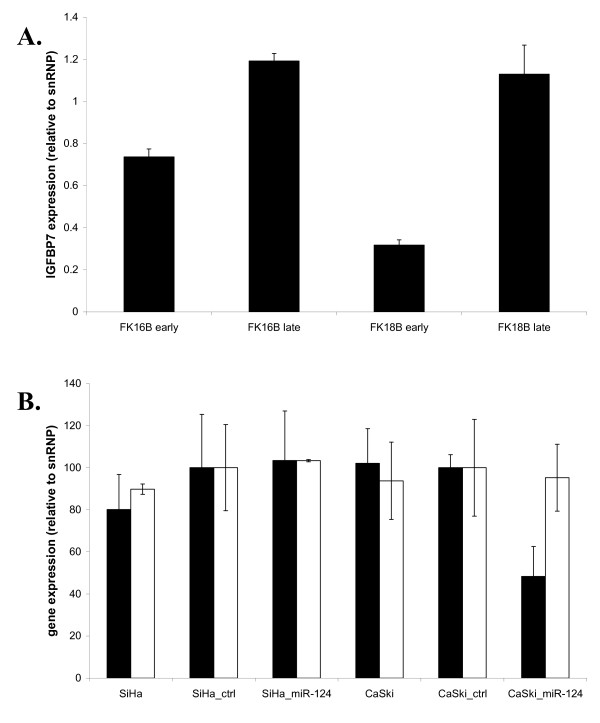
***IGFBP7* and *SLC25A36* expression in HPV-immortalised cells and cervical cancer cells transduced with *hsa-miR-124*. A.** Expression levels of *IGFBP7*, a potential target gene of *hsa-miR-124*, were increased in late passages of FK16B and FK18B cells, also showing increased methylation of *hsa-miR-124*, compared to their corresponding early passages. **B**. Effects of ectopic *hsa-miR-124 *expression on mRNA expression of *IGFBP7 *(grey bars) in SiHa and CaSki cells. Expression of *SLC25A36 *(white bars), a gene without an *hsa-miR-124 *target site, was also determined as a control. Expression in CaSki_ctrl and SiHa_ctrl was set to 100%, respectively. Results from 3 independent experiments showed that *IGFBP7 *expression was decreased in CaSki_miR-124 but not in SiHa_miR-124 cells compared to their parental and empty vector control cell lines. Expression of *SLC25A36 *was similar in cells with and without ectopic *hsa-miR-124 *expression.

Quantitative RT-PCR was performed for *IGFBP7 *in early and late passages of FK16B and FK18B cells as well as SiHa and CaSki cells with and without ectopic *hsa-miR-124 *expression, to determine the effects of *hsa-miR-124 *on the mRNA level of *IGFBP7*. Interestingly, late passages of FK16B and FK18B cells, which were shown to have increased levels of hsa-miR-124-1 and hsa-miR-124-2 methylation and decreased levels of *hsa-miR-124 *expression (Figure 2), showed increased levels of *IGFBP7 *expression compared to their corresponding early passages (Figure 4A). In CaSki cells ectopic *hsa-miR-124 *expression resulted in reduction of *IGFBP7 *expression of more than 50% compared to the empty vector control cells and parental cells, however, no effect was observed in SiHa_miR-124 cells. As a control, *SLC25A36*, a gene without any *hsa-miR-124 *target sites, was included in this analysis and showed similar expression levels in cells with and without ectopic *hsa-miR-124 *expression (Figure 4B).

These results indicate that *IGFBP7 *may be a target of *hsa-miR-124 *in part of the cervical carcinomas.

### *hsa-miR-124 *methylation and silencing is frequent in cervical (pre)malignant lesions

**Figure 5:**
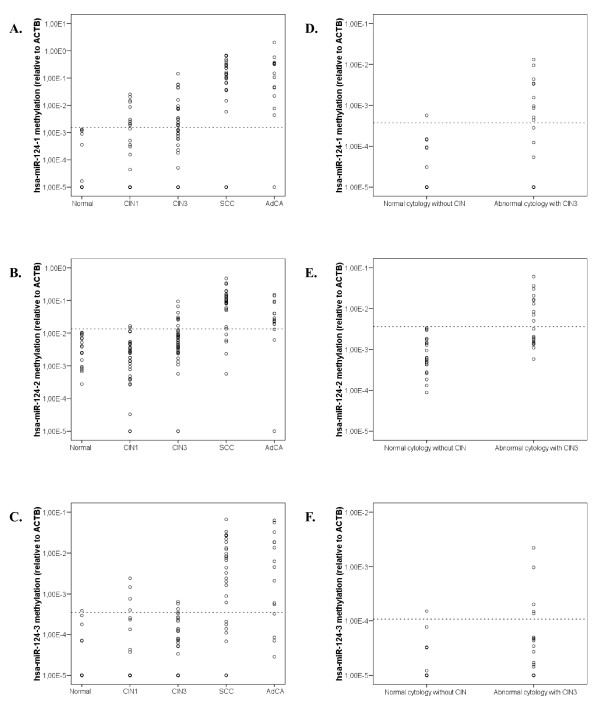
***Hsa-miR-124* methylation levels in cervical specimens**. Dotplots showing the levels of methylation for **A.** hsa-miR-124-1, **B.** hsa-miR-124-2 and C. hsa-miR-124-3 in normal cervical specimens, CIN1 lesions, CIN3 lesions, SCCs and AdCAs. Methylation levels in cervical scrapes of women with normal cytology without underlying CIN and women with abnormal cytology with underlying CIN3 lesions are shown in **D**. for hsa-miR-124-1, **E**. for hsa-miR-124-2 and **F**. for hsa-miR-124-3.

Since methylation of *hsa-miR-124 *becomes detectable in late passages of HPV-immortalised keratinocytes mimicking premalignant cervical disease [33], these regions may provide markers for the detection of cervical cancer and its high-grade precursor lesions. We therefore determined the methylation levels of all promoter regions in 18 normal cervical specimens, 36 CIN1 lesions, 41 CIN3 lesions, 29 SCCs and 15 AdCAs (Figure 5A-C). Using a cut off value based on the 99% confidence interval of the mean value measured in the normal specimens, only 1 normal sample (5.6%) scored positive for hsa-miR-124-3 methylation, whereas no normal samples scored positive for hsa-miR-124-1 and hsa-miR-124-2 methylation. For hsa-miR-124-1 and hsa-miR-124-2, respectively, the percentages of methylation positivity increased from 27.8% and 5.6% in CIN1, to 46.3% and 19.5% in CIN3, to 86.2% and 82.8% in SCCs. In addition, high percentages of methylation positivity for hsa-miR-124-1 and hsa-miR-124-2 were found in AdCAs as well (93.3% and 80% respectively). In concordance with the fact that *in vitro *hsa-miR-124-3 was only methylated in cervical cancer cell lines and not in HPV-immortalised precursor cell lines, the frequency of hsa-miR-124-3 methylation was low in CIN1 (11.1%) and CIN3 (9.8%) lesions, but increased to 72.4% and 73.3% in SCCs and AdCAs, respectively (Table 2).

**Table 2:** Frequencies of *hsa-miR-124* methylation detected by qMSP analysis

	methylation positivity (%)			Combined (and/or) methylation positivity (%)			
	hsa-miR-124-1	hsa-miR-124-2	hsa-miR-124-3	hsa-miR-124-1/-2/-3	hsa-miR-124-1/-2	hsa-miR-124-1/-3	hsa-miR-124-2/-3
Normal (n = 18)	0.0	0.0	5.6	5.6	0.0	5.6	5.6
CIN1 (n = 36)	27.8	5.6	11.1	30.6	30.6	27.8	13.9
CIN3 (n = 41)	46.3	19.5	9.8	58.5	58.5	48.8	26.8
SCC (n = 29)	86.2	82.8	72.4	93.1	93.1	89.7	86.2
AdCA (n = 15)	93.3	80.0	73.3	100.0	93.3	100.0	93.3

normal cytology without CIN (n = 22)	4.5	0.0	4.5	9.1	4.5	9.1	4.5
abnormal cytology with CIN3 (n = 21)	47.6	47.6	23.8	71.4	71.4	57.1	47.6

A combined scoring system for hsa-miR-124-1 and/or hsa-miR-124-2 methylation resulted in 0% positivity in normal cervix, 30.6% in CIN1 lesions, 58.5% in CIN3 lesions and 93.1% in SCCs (Table 2). The difference in methylation frequency between normal samples and low-grade lesions (CIN1) on one hand and high-grade lesions (CIN3) and SCCs on the other hand was highly significant (p < 0.001). Addition of hsa-miR-124-3 methylation resulted in the detection of one extra AdCA as well as one extra normal specimen.

**Figure 6:**
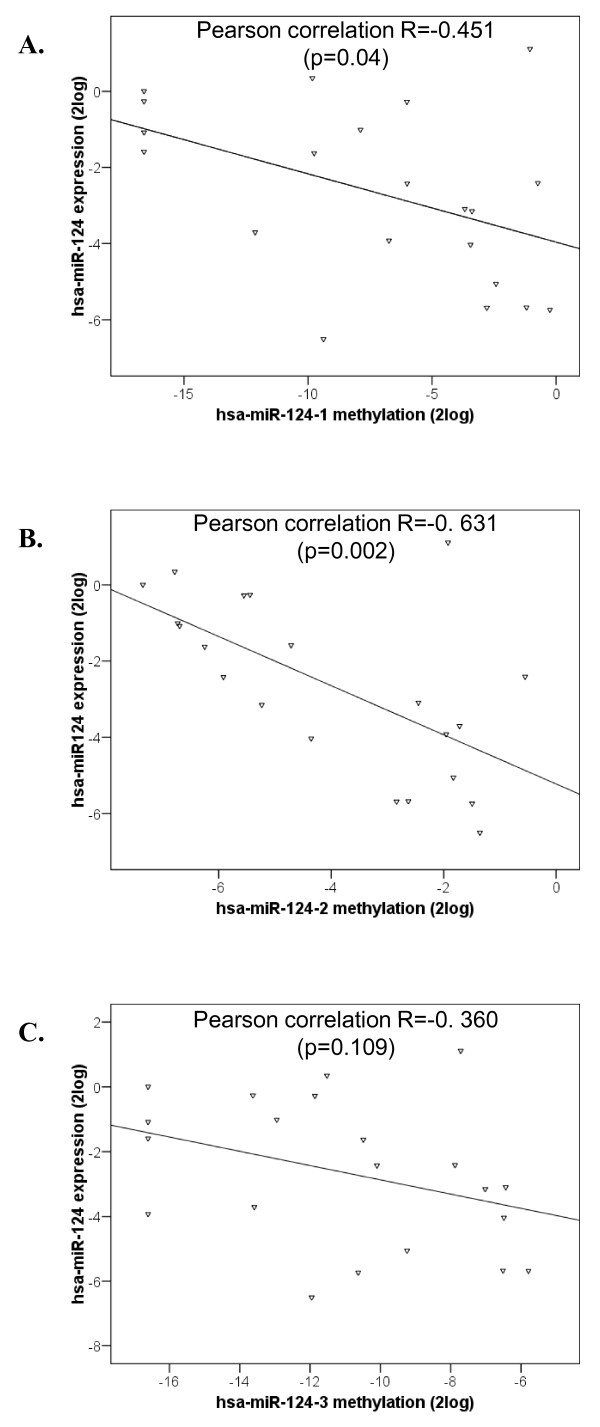
**Correlation between hsa-miR-124 methylation and expression in cervical tissue specimens. **The overall correlation between **A.** hsa-miR-124-1, **B.** hsa-miR-124-2 and **C.** hsa-miR-124-3 methylation levels and *hsa-miR-124 *expression in CIN2/3 lesions, SCCs and AdCAs is shown.

To determine whether *hsa-miR-124 *methylation also resulted in silencing of *hsa-miR-124 *expression in cervical lesions, we measured the expression of *hsa-miR-124 *in a panel of frozen specimens of normal cervical squamous epithelium (n = 5), CIN2/3 (n = 7), cervical SCCs (n = 9) and AdCAs (n = 5). To eliminate the possibility of confounding results due to stromal expression, all samples were microdissected. The average expression of *hsa-miR-124 *in CIN2/3 lesions and cervical carcinomas compared to normal cervical epithelium was 4.4 fold decreased (p = 0.001). In addition, we determined the correlation between *hsa-miR-124 *expression and methylation levels for the 3 regions in CIN2/3 lesions and carcinomas (Figure 6). Methylation levels of hsa-miR-124-1 and hsa-miR-124-2 were significantly negatively correlated with *hsa-miR-124 *expression levels (R = -0.451, p = 0.04 and R = -0.631, p = 0.002, respectively), whereas for hsa-miR-124-3 no significant correlation was found (R = -0.360, p = 0.109).

### *Hsa-miR-124 *methylation in cervical scrapes is predictive of underlying lesions

To be considered as a candidate disease marker that potentially could be of value for the detection of high-grade CIN and carcinoma in cervical screening, methylation of *hsa-miR-124 *should be detectable in cervical scrapes containing few abnormal cells in a background of normal cells. As a proof of principle we analysed the methylation levels of all 3 loci in 22 hrHPV-positive cytologically normal cervical scrapes of women without evidence of CIN disease in the subsequent 5 years and 21 hrHPV-positive cytologically abnormal scrapes of women with CIN3, diagnosed within 18 months of follow-up (Figure 5D-F). Using the same analysis method as described above, we found methylation of hsa-miR-124-1 and/or hsa-miR-124-2 in 4.5% (1/22) of the women without disease versus 71.4% (15/21) of women with CIN3. Methylation analysis of hsa-miR-124-3 had no additive value in this sample series (Table 2).

Collectively, these results show that methylation analysis of hsa-miR-124-1 and hsa-miR-124-2 provides an attractive candidate marker for the triage of hrHPV-positive women.

## Discussion

In this study we showed that epigenetic silencing of *hsa-miR-124 *is functionally involved in cervical carcinogenesis and may provide a valuable marker for risk stratification of hrHPV-positive women. Using qMSP analysis, we found methylation of hsa-miR-124-1 and/or hsa-miR-124-2 in none of the normal tissues, 58.5% of CIN3 lesions, 93.1% of SCCs and 93.3% of AdCAs. Increased methylation levels of hsa-miR-124-1 and hsa-miR-124-2 in cervical tissue specimens were significantly correlated with lower *hsa-miR-124 *expression levels. Analysis of cervical scrapes showed that only 4.5% of hrHPV-positive scrapes without CIN disease in follow-up was positive compared to 71.4% of hrHPV-positive scrapes with CIN3 in follow-up. To the best of our knowledge this study provides the first evidence of DNA methylation-based silencing of a miRNA in cervical cancer.

Methylation of *hsa-miR-124 *was found in cervical cancer cell lines SiHa, CaSki and HeLa as well as in late passages of HPV16/18 immortalised keratinocytes, reminiscent of high grade cervical precursor lesions, but not in normal primary keratinocytes. The fact that methylation of *hsa-miR-124 *and concomitant reduced *hsa-miR-124 *expression was found in late passages of HPV-immortalised keratinocytes but not in early passages, indicates that this event takes place post-immortalisation and is not directly related to the presence of hrHPV. Ectopic expression of *hsa-miR-124 *decreased the proliferation rate of both SiHa and CaSki cells and also inhibited the migratory capacity of SiHa cells. Effects on the migratory capacity of CaSki cells were difficult to ascertain due to the specific growth characteristics of this cell line. Consistent with our findings, both Agirre *et al *and Furuta *et al *observed inhibitory effects on cellular growth upon reintroduction of *hsa-miR-124 *expression in acute lymphoblastic leukaemia (ALL)-derived cells and hepatocellular carcinoma cell lines [26,32].

One of the previously identified targets mediating the tumour suppressive function of *hsa-miR-124 *is *CDK6*, via *CDK6 *mediated phosphorylation and subsequent inactivation of the tumour suppressor *pRb *[29,32]. However, in cervical cancer the virally encoded oncoprotein E7 is thought to bind and inactivate *pRb*, suggesting *hsa-miR-124 *may (partly) function via other targets in this type of cancer. Using data from a recent study by Baek *et al*, we identified *IGFBP7 *as a promising target gene in cervical cancer [17]. *IGFBP7 *mRNA levels were increased in late passages of FK16B and FK18B cells compared to their corresponding early passages, which also showed increased levels of hsa-miR-124-1 and hsa-miR-124-2 methylation and decreased levels of *hsa-miR-124 *expression. In addition, *IGFBP7 *showed decreased mRNA expression in CaSki cells ectopically expressing *hsa-miR-124 *compared to empty vector control cells and parental cells. No effect was seen in SiHa_miR-124 cells, however. These results indicate that *IGFBP7 *may be a potential target of *hsa-miR-124 *in part of the cervical cancers, but other targets may be relevant for the tumour suppressive function of *hsa-miR-124 *in cervical cancer as well. *IGFBP7 *is part of the insulin-like growth factor (IGF) axis, which has been implicated in cervical cancer before. It was shown that the IGF-axis may influence the persistence of hrHPV infections and that abnormally balanced co-expression of IGFBP family members is associated with gynaecological malignancy [42,43]. *IGFBP7 *is the only member of the IFGBP family that binds insulin instead of IGF and is relatively unknown compared to its family members. On one hand *IGFBP7 *has recently been described as a tumour suppressor gene in colorectal cancer and was shown to induce senescence in cells harbouring oncogenic BRAF [44-46], whereas on the other hand *IGFBP7 *was shown to have oncogenic properties in gliomas [47]. Further functional studies are needed to investigate whether the tumour suppressive function of *hsa-miR-124 *in cervical cancer may in part be mediated via *IGFBP7*.

*Hsa-miR-124 *was originally described as a brain-specific miRNA, involved in neuronal differentiation. Lujambio *et al *were the first to show methylation-mediated silencing of *hsa-miR-124 *in different human cancer types, reaching the highest frequency in colorectal cancer (75%) [29]. In acute lymphoblastic leukaemia (ALL) methylation of *hsa-miR-124 *was shown to negatively affect clinical outcome [30,32]. In ALL hsa-miR-124-1 showed the highest frequency of methylation as was also found for cervical cancer in our study. Interestingly, in gastric cancer and hepatocellular carcinoma, hsa-miR-124-3 showed the highest frequency of methylation [25,26], whereas in ALL and cervical cancer hsa-miR-124-3 showed the lowest frequencies of methylation positivity. This may indicate that hsa-miR-124-3 methylation is tissue or tumour type dependent. The fact that hsa-miR-124-3 methylation was infrequent in cervical precursor lesions and HPV-immortalised keratinocyte cell lines supports the notion that at least in cervical carcinogenesis hsa- miR-124-3 methylation is a rather late event. Overall, the methylation positivity rates for *hsa-miR-124 *found in our study rank among the highest currently reported, although the use of different assays and methods, including non-quantitative MSP and combined bisulfite restriction analysis, makes a direct comparison difficult. Importantly, *hsa-miR-124 *methylation in cervical cancer was histotype-independent and could already be detected in CIN3 lesions and scrapes of women with underlying CIN3, underlining its potential value for cervical cancer screening.

## Conclusions

This study shows that methylation of *hsa-miR-124 *is a frequent and functionally relevant event in cervical carcinogenesis. The high positivity rates in CIN3 lesions and carcinomas as well as in scrapes with underlying CIN3 lesions combined with the fact that *hsa-miR-124 *methylation is not directly related to the presence of hrHPV, indicate that *hsa-miR-124 *methylation may provide a valuable marker for the triage of hrHPV-positive women. Future studies in large population-based cohorts will determine whether testing for *hsa-miR-124 *methylation either or not in combination with other promising methylation markers such as CADM1 and MAL [13,14] can improve future cervical screening strategies based on primary hrHPV testing.

## Competing interests

The authors declare that they have no competing interests.

## Authors' contributions

SMW and RAAvB performed all experiments and data analysis and drafted the manuscript. RDMS and PJFS participated in the design of the study and the drafting of the manuscript. FEH, RA and ClS greatly contributed to the functional experiments with retroviral constructs. CJLM, BD and GAM contributed to the conception of the study and critically revised the manuscript. All authors read and approved the final manuscript.

## Supplementary Material

Additional file 1 **HPV typing results of clinical specimens included in this study**. In this additional table, for all clinical specimens included in this study, HPV typing results by general primer GP5+/6+ PCR and reverse line blot are given. Click here for file
