# Supplementary material for: Methylation-mediated silencing and tumour suppressive function of hsa-miR-124 in cervical cancer
Source: Mol Cancer. 2010 Jun 26;9:167. doi: 10.1186/1476-4598-9-167 (PMC2917428; doi:10.1186/1476-4598-9-167)
Supplement: Additional file 1 — HPV typing results of clinical specimens included in this study. In this additional table, for all clinical specimens included in this study, HPV typing results by general primer GP5+/6+ PCR and reverse line blot are given. [file 1476-4598-9-167-S1.DOC]

**Supplementary Table 1.** HPV genotyping of specimens included in this study

| **sample ID** | **histology/cytology** | **HPV type** |
| --- | --- | --- |
| FFPE_1 | Normal | neg |
| FFPE_2 | Normal | neg |
| FFPE_3 | Normal | neg |
| FFPE_4 | Normal | neg |
| FFPE_5 | Normal | neg |
| FFPE_6 | Normal | neg |
| FFPE_7 | Normal | neg |
| FFPE_8 | Normal | neg |
| FFPE_9 | Normal | neg |
| FFPE_10 | Normal | neg |
| FFPE_11 | Normal | neg |
| FFPE_12 | Normal | neg |
| FFPE_13 | Normal | neg |
| FFPE_14 | Normal | neg |
| FFPE_15 | Normal | neg |
| FFPE_16 | Normal | neg |
| FFPE_17 | Normal | neg |
| FFPE_18 | Normal | neg |
| FFPE_19 | CIN1 | 51 |
| FFPE_20 | CIN1 | neg |
| FFPE_21 | CIN1 | neg |
| FFPE_22 | CIN1 | neg |
| FFPE_23 | CIN1 | neg |
| FFPE_24 | CIN1 | neg |
| FFPE_25 | CIN1 | neg |
| FFPE_26 | CIN1 | neg |
| FFPE_27 | CIN1 | neg |
| FFPE_28 | CIN1 | 56 |
| FFPE_29 | CIN1 | neg |
| FFPE_30 | CIN1 | 16 |
| FFPE_31 | CIN1 | neg |
| FFPE_32 | CIN1 | neg |
| FFPE_33 | CIN1 | 82 |
| FFPE_34 | CIN1 | X |
| FFPE_35 | CIN1 | neg |
| FFPE_36 | CIN1 | neg |
| FFPE_37 | CIN1 | neg |
| FFPE_38 | CIN1 | neg |
| FFPE_39 | CIN1 | neg |
| FFPE_40 | CIN1 | 59 |
| FFPE_41 | CIN1 | neg |
| FFPE_42 | CIN1 | 58 |
| FFPE_43 | CIN1 | neg |
| FFPE_44 | CIN1 | neg |
| FFPE_45 | CIN1 | 66 |
| FFPE_46 | CIN1 | neg |
| FFPE_47 | CIN1 | X |
| FFPE_48 | CIN1 | neg |
| FFPE_49 | CIN1 | X |
| FFPE_50 | CIN1 | neg |
| FFPE_51 | CIN1 | 33 |
| FFPE_52 | CIN1 | X |
| FFPE_53 | CIN1 | neg |
| FFPE_54 | CIN1 | neg |
| FFPE_55 | CIN3 | 73 |
| FFPE_56 | CIN3 | 51 |
| FFPE_57 | CIN3 | 16 |
| FFPE_58 | CIN3 | 33 |
| FFPE_59 | CIN3 | 16 |
| FFPE_60 | CIN3 | 16 |
| FFPE_61 | CIN3 | 31 |
| FFPE_62 | CIN3 | 52 |
| FFPE_63 | CIN3 | 26 |
| FFPE_64 | CIN3 | 16 |
| FFPE_65 | CIN3 | 16 |
| FFPE_66 | CIN3 | 16 |
| FFPE_67 | CIN3 | neg |
| FFPE_68 | CIN3 | X |
| FFPE_69 | CIN3 | 31 |
| FFPE_70 | CIN3 | 18 |
| FFPE_71 | CIN3 | 82 |
| FFPE_72 | CIN3 | 16 |
| FFPE_73 | CIN3 | 16 |
| FFPE_74 | CIN3 | 16 |
| FFPE_75 | CIN3 | 16 |
| FFPE_76 | CIN3 | 16 |
| FFPE_77 | CIN3 | 16 |
| FFPE_78 | CIN3 | 31 |
| FFPE_79 | CIN3 | 16 |
| FFPE_80 | CIN3 | 16 |
| FFPE_81 | CIN3 | 16 |
| FFPE_82 | CIN3 | 16 |
| FFPE_83 | CIN3 | 16 |
| FFPE_84 | CIN3 | X |
| FFPE_85 | CIN3 | 51 |
| FFPE_86 | CIN3 | 18 |
| FFPE_87 | CIN3 | 33 |
| FFPE_88 | CIN3 | 16 |
| FFPE_89 | CIN3 | 16 |
| FFPE_90 | CIN3 | 16 |
| FFPE_91 | CIN3 | 16 |
| FFPE_92 | CIN3 | 16 |
| FFPE_93 | CIN3 | 16 |
| FFPE_94 | CIN3 | 35 |
| FFPE_95 | CIN3 | 16 |
| FFPE_96 | SCC | 70;68;39 |
| FFPE_97 | SCC | 56 |
| FFPE_98 | SCC | 16 |
| FFPE_99 | SCC | 16 |
| FFPE_100 | SCC | 45 |
| FFPE_101 | SCC | 16 |
| FFPE_102 | SCC | 16;51 |
| FFPE_103 | SCC | 16;33 |
| FFPE_104 | SCC | 51 |
| FFPE_105 | SCC | 52 |
| FFPE_106 | SCC | 16 |
| FFPE_107 | SCC | 16 |
| FFPE_108 | SCC | 16 |
| FFPE_109 | SCC | 16 |
| FFPE_110 | SCC | 16 |
| FFPE_111 | SCC | 16 |
| FFPE_112 | SCC | 16 |
| FFPE_113 | SCC | 18 |
| FFPE_114 | SCC | 16 |
| FFPE_115 | SCC | 33 |
| FFPE_116 | SCC | 16 |
| FFPE_117 | SCC | 16 |
| FFPE_118 | SCC | 16 |
| FFPE_119 | SCC | 16 |
| FFPE_120 | SCC | 16 |
| FFPE_121 | SCC | 16 |
| FFPE_122 | SCC | 18 |
| FFPE_123 | SCC | 39 |
| FFPE_124 | SCC | 16 |
| FFPE_125 | AdCA | 16 |
| FFPE_126 | AdCA | 18 |
| FFPE_127 | AdCA | 18 |
| FFPE_128 | AdCA | 18 |
| FFPE_129 | AdCA | 33;39 |
| FFPE_130 | AdCA | 45 |
| FFPE_131 | AdCA | 18 |
| FFPE_132 | AdCA | 16 |
| FFPE_133 | AdCA | 18 |
| FFPE_134 | AdCA | 16 |
| FFPE_135 | AdCA | 16 |
| FFPE_136 | AdCA | 16 |
| FFPE_137 | AdCA | 18 |
| FFPE_138 | AdCA | 18 |
| FFPE_139 | AdCA | 18 |
| scrape_1 | normal | 56;84 |
| scrape_2 | normal | ntd |
| scrape_3 | normal | X |
| scrape_4 | normal | 66 |
| scrape_5 | normal | 66 |
| scrape_6 | normal | 56 |
| scrape_7 | normal | 16 |
| scrape_8 | normal | 16 |
| scrape_9 | normal | 52;89 |
| scrape_10 | normal | 16;18 |
| scrape_11 | normal | 31;45 |
| scrape_12 | normal | 66 |
| scrape_13 | normal | 18 |
| scrape_14 | normal | X |
| scrape_15 | normal | 31 |
| scrape_16 | normal | 33 |
| scrape_17 | normal | X |
| scrape_18 | normal | 16 |
| scrape_19 | normal | 16 |
| scrape_20 | normal | 39;67 |
| scrape_21 | normal | 51 |
| scrape_22 | normal | 45 |
| scrape_23 | severe dyskaryosis or worse | 16 |
| scrape_24 | severe dyskaryosis or worse | 16 |
| scrape_25 | severe dyskaryosis or worse | 39 |
| scrape_26 | severe dyskaryosis or worse | 16 |
| scrape_27 | severe dyskaryosis or worse | 16 |
| scrape_28 | severe dyskaryosis or worse | 16 |
| scrape_29 | severe dyskaryosis or worse | 16 |
| scrape_30 | severe dyskaryosis or worse | 18 |
| scrape_31 | severe dyskaryosis or worse | ntd |
| scrape_32 | severe dyskaryosis or worse | 16 |
| scrape_33 | severe dyskaryosis or worse | 16;18 |
| scrape_34 | severe dyskaryosis or worse | 16 |
| scrape_35 | severe dyskaryosis or worse | 16;81 |
| scrape_36 | severe dyskaryosis or worse | 16 |
| scrape_37 | severe dyskaryosis or worse | 33 |
| scrape_38 | severe dyskaryosis or worse | 58 |
| scrape_39 | severe dyskaryosis or worse | 31 |
| scrape_40 | severe dyskaryosis or worse | 16;31 |
| scrape_41 | severe dyskaryosis or worse | 33;39 |
| scrape_42 | severe dyskaryosis or worse | 16 |
| scrape_43 | severe dyskaryosis or worse | 16 |

CIN: cervical intraepithelial neoplasia; SCC: squamous cell carcinoma; AdCA: adenocarcinoma; neg: no HPV detected; NTD: not to determine due to negative housekeeping gene (i.e. beta-globin) control; X: HPV type(s) detected by enzyme immunoassay but not by reverse line blot genotyping, indicating a type, sub-type or variant not detectable with probes used for reverse line blot hybridization
